# Predictive Chromatography of Leaf Extracts Through Encoded Environmental Forcing on Phytochemical Synthesis

**DOI:** 10.3389/fpls.2021.613507

**Published:** 2021-08-25

**Authors:** Junelle Rey C. Bacong, Drandreb Earl O. Juanico

**Affiliations:** DataSc/ense TechnoCoRe, Technological Institute of the Philippines, Quezon City, Philippines

**Keywords:** plant-environment interactions, phenotypic plasticity, phytochemical profiling, plant therapeutics, plant extracts, *Blumea balsamifera*, convolutional neural network

## Abstract

Environment fluctuations can influence a plant's phytochemical profile via phenotypic plasticity. This adaptive response ensures a plant's survival under fluctuating growth conditions. However, the resulting plant extract composition becomes unpredictable, which is a problem for highly standardized medicinal applications. Here we demonstrate, for the first time, the feasibility of tracking the changes in the phytochemical profile based on real-time measurements of a few environment and extract-preparation variables. As a result, we predicted the chromatograms of *Blumea balsamifera* extracts through an imputation-augmented convolutional neural network, which uses the image-transformed temporal measurements of the variables. We developed a sensor network that collected data in a greenhouse and a training algorithm that concurrently generated a data representation of the implicit plant-environment interactions leading to the mutable chromatograms of leaf extracts. We anticipate the generic applicability of the method for any plant and recognize its potential for addressing the standardization problems in plant therapeutics.

## Introduction

Plants may be thought of as factories that synthesize highly complex and unusual substances for various medical and non-medical applications (Mishra and Tiwari, 2011; Nikam et al., 2012). These complex phytochemical mixtures in herbal or plant-derived medicines have been shown to have advantages over the single molecules that are isolated or synthetically modified from natural sources (Rodriguez-Concepcion et al., 2006; Carmona and Soares Pereira, 2013; Ekor, 2014). This has led to a tremendous increase in the use of herbal products and supplements over the past three decades, as many people around the world have resorted to using these products for treating various health-related concerns (Calixto, 2000; WHO, 2004; Ekor, 2014). However, the production of herbal medicines is a gradual and meticulous process. It involves three basic steps: (i) identification of herbs based on macroscopical and microscopical features; (ii) evaluation of drugs for the confirmation of their identity and purity; and (iii) standardization (Kunle et al., 2012; Newmaster et al., 2013). The standardization of herbal formulations encompasses all of the quality control measures taken during the manufacturing process such as sample preparation and phytochemical evaluation, as well as microbial, biological, and toxicity testing (Calixto, 2000; Rodriguez-Concepcion et al., 2006; Kunle et al., 2012; Newmaster et al., 2013). Additionally, guidelines and protocols are utilized to ensure the safety, quality, and efficacy of all herbal products and formulations (WHO, 1998; Harvey, 2008; Sahoo et al., 2010; Newmaster et al., 2013).

In the Philippines, there are 10 herbal plants that are recommended by the Department of Health for medical applications and potential product commercialization. These 10 medicinal plants have already been scientifically and clinically validated. In fact, these plants are listed under the Republic Act No. 8423 and by the Philippine Institute of Traditional and Alternative Health Care as recommended for use in treating specific physiological problems (Ammakiw and Odiem, 2013; Boy et al., 2018). An example of which is *Blumea balsamifera* (locally known and referred to hereafter as “sambong”), is a shrub that grows across Southeast Asia, India, and China, known for managing urolithiasis and other kidney problems (Ammakiw and Odiem, 2013; Montealegre and De Leon, 2017; Boy et al., 2018). However, despite proven therapeutic effects, the herbal products derived from such medicinal plants remain difficult to commercialize because of the inconsistent use of extraction methods and the variable content in different batches of these herbal formulations (Sahoo et al., 2010; Carmona and Soares Pereira, 2013). As such, the primary goal of the standardization of herbal formulations is to ensure a reproducible quality of herbal products (Calixto, 2000; Rodriguez-Concepcion et al., 2006; Sahoo et al., 2010; Kunle et al., 2012).

A very important aspect in the standardization of plant-derived medicinal products is the phytochemical evaluation. This involves the identification and relative quantification of bioactive compounds in the herbal extracts. The evaluation is conducted by analyzing the phytochemical profile of the extracts obtained from tedious chromatographic and spectroscopic procedures. Such procedures involve the use of highly-technical setups such as liquid and gas chromatography in conjunction with mass or ultraviolet spectroscopy (LC/GC-MS, LC/GC-UV), capillary electrophoresis, nuclear magnetic resonance spectral analysis, attenuated total reflection, and Fourier transform infrared spectroscopic imaging, among others (Dias et al., 2012; Seger et al., 2013; Huck, 2015). However, despite the use of these modern chemical and analytical procedures, the determination and isolation of bioactive metabolites in plant materials remains challenging (Calixto, 2000). Such difficulty arises from the plants' inherent phenotypic plasticity in response to stress and their environment, resulting to significant variability in their phytochemical make-up. For instance, raw herbal materials cultivated and collected from the same area of vegetation may have different phytochemical profiles and may thereby exhibit different bioactivities. Pérez-Balibrea et al. (2008) showed that the light treatment of sprouting broccoli (*Brassicaceae*) seeds increases the concentration of health-promoting phytochemicals, such as vitamin C, glucosinolates, and phenolic compounds. Odjegba and Alokolaro (2013) simulated the effects of a drought and varying salinity conditions in *Acalypha wilkesiana* plants, which resulted in a decrease in the quantity of alkaloids, flavonoids, and tannins in the extracts, as well as an increase in the saponin production levels. Due to their plasticity, plants can adjust their responses to a multitude of biotic and abiotic stresses. Therefore, changes in environmental conditions such as temperature, humidity, sunlight, rainfall, and soil conditions, as well as diurnal and seasonal cycles, can promote significant variability in the phytochemical make-up of raw herbal materials.

The complex nature of plant extracts makes the development of herbal products a difficult task. A large analytical effort and high-quality manufacturing skills are needed to produce standardized and quality controlled herbal formulations (Cravotto et al., 2010; Carmona and Soares Pereira, 2013). One approach to studying the complexity of these plant extracts is through chemometrics, which aims to understand metabolomic or chromatographic data using multivariate data analysis (Parker et al., 2009; Turi et al., 2015). Chemomemtric analysis denotes the application of statistical tools such as principal component analysis (PCA) (Le Gall et al., 2003; Want et al., 2010; Worley and Powers, 2013; Wolfender et al., 2015), support vector machines (SVMs) (Zheng et al., 2009; Gromski et al., 2015), and multivariate regression models (Brown et al., 2012; Das et al., 2017; Ballesteros-Vivas et al., 2019) to examine and validate the phytochemistry of organic extracts based on their chromatographic or metabolomic profiles. Unsupervised analytical techniques such as PCA and SVMs have been used to determine the secondary metabolites that contribute to the specific bioactivity of a plant extract (Le Gall et al., 2003; Zheng et al., 2009; Want et al., 2010; Worley and Powers, 2013; Gromski et al., 2015; Wolfender et al., 2015). Multivariate regression models, a type of supervised statistical technique, have been used to correlate the extraction parameters, such as the solvent type and pH, with the concentrations of specific metabolites in the plant extracts (Brown et al., 2012; Das et al., 2017; Ballesteros-Vivas et al., 2019).

However, these types of chemometric tools usually require the cumbersome process of choosing specific features that may be suboptimal for a given task. Artificial intelligence technologies such as deep learning (LeCun et al., 2015; Schmidhuber, 2015) have generated new methods over recent years that permit the determination of the most suitable set of features within the training process, without any involvement from the investigator (Zhang et al., 2017). In natural product research wherein the volume of data sets is typically very large, deep learning methodologies have shown promising results (Chen et al., 2018; Sarker and Nahar, 2018). For instance, artificial neural networks (ANNs) (Dahmoune et al., 2015; Eftekhari et al., 2018) were trained to determine the non-linear relationship between the laboratory and extraction parameters as the inputs and the metabolite concentrations as the outputs. ANNs were also used to predict the bioactivity of plant extracts given the relative concentration of their secondary metabolites (Hosu et al., 2014; Das et al., 2017). Moreover, convolutional neural networks (CNNs) that are typically used for extracting features and classifying spatial and grid-structured data such as images have been applied to the 2D HSQC spectra of compounds from marine and terrestrial organisms for the characterization of their metabolic profiles (Zhang et al., 2017; Reher et al., 2020). This particular CNN tool leverages the wealth of these spectral data sets constructed over the past four decades from natural product research (Zhang et al., 2017). CNNs were also used to analyze LC-MS data, particularly in classifying the true and false peaks in the LC-MS spectra (Kantz et al., 2019). The cumbersome process does not justify the prediction performance of these chemometric tools.

A missing piece of information is likely the source of the unexplained variability in existing predictive techniques applied to the chromatographic characterization of plants. Few studies have accounted for the gross effects of the environment on the phytochemical compositions of herbal extracts, which also makes the standardization of herbal formulations difficult to achieve. Most previous studies have focused primarily on characterizing specific groups of phytochemicals, such as phenolic compounds, and their related bioactivity in the extracts (Le Gall et al., 2003; Zheng et al., 2009; Want et al., 2010; Brown et al., 2012; Worley and Powers, 2013; Dahmoune et al., 2015; Gromski et al., 2015; Wolfender et al., 2015; Das et al., 2017; Eftekhari et al., 2018; Ballesteros-Vivas et al., 2019). In this work, we present a novel method for predicting the chromatogram of sambong leaf extracts using sensor data collected from the environment in which the plant has been exposed for over 1 month. We used deep learning technology, particularly CNNs, to correlate the abiotic stresses, such as the changes in temperature, humidity, ambient light, soil pH, and soil moisture, with the supposed chromatograms of the leaf extracts. Herein, we show that the environmental forcing on phytochemical synthesis can be encoded using CNNs. As a result, the trained network model can be used to accurately predict the entire chromatographic profile of plant extracts based on different time-varying environmental parameters, as well as using the controlled laboratory variables. Unlike previous studies that have focused only on analyzing specific groups of compounds, our work predicts the entire phytochemical profile that represents the synergistic contributions of each putative metabolite in the extract. As such, this method can be used to evaluate the phytochemical composition of herbal extracts without undergoing tedious laboratory and chromatographic procedures. Our work on predictive chromatography offers a fast, accurate, and high-throughput alternative for phytochemical evaluation, which is an integral component of standardizing herbal formulations. To our knowledge, this study is the first to consider the extraction of temporal information from environmental data using CNNs to predict the chromatogram of a plant extract.

## Materials and Methods

**Figure 1:**
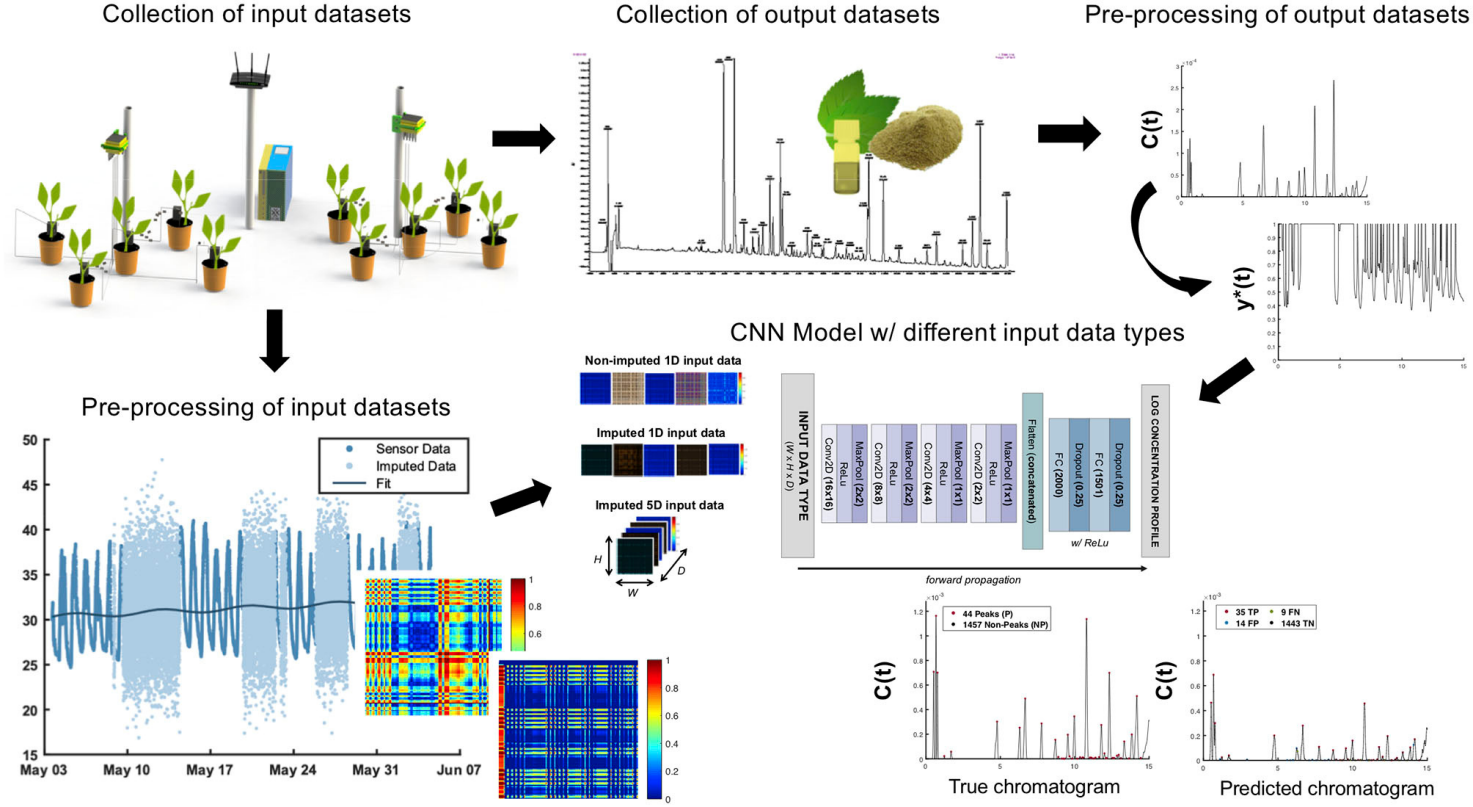
A method of predicting the phytochemical profile of plant extracts. Predictive chromatography of sambong leaf extracts obtained using the environmental parameters such as temperature and humidity, ambient light, soil moisture, and soil pH. The training of the CNN model proceeds from the collection and pre-processing of input and output data sets obtained from REMS and LC-UV chromatography, respectively. Using the images of the environmental time-series data as inputs, the CNN model will be able to predict the relative percentage concentration profile of an extract taken from a specific sambong plant.

The underlying workflow of the predictive chromatography is graphically outlined in Figure 1. The following methods are comprised of separate data collections for input and output data sets. Subsequent pre-processing procedures were applied to both the input and output data sets prior to the neural network training and model evaluation.

### Collection of Input Data Sets

An in-house remote environmental monitoring system (REMS) was installed in Los Baños, Laguna to monitor and record the real-time data for soil pH, soil moisture, ambient temperature, relative humidity, and light intensity over a 1-month study period (see Supplementary Figures 1, 2). The REMS consists of a plurality of sensing instruments that are made from off-the-shelf sensors and meters for detecting temperature and humidity (DHT22), soil moisture (DFRobot SKU SEN0193), ambient light (Adafruit TSL2591), and soil pH (Fisher Science Education PH700 Rapitest pH meter). These instruments are linked together via an expansion port that facilitates data transmission from the sensors. The aggregated environmental data from the linked instruments are then sent to a database server.

### Collection of Output Data Sets

#### Chemicals

The naringenin standards were sourced from Sigma-Aldrich. The solvents used for extraction, namely ethyl acetate, methanol, and n-hexane, were all HPLC grade and were obtained from RCI Labscan. LC-MS-grade methanol, formic acid and acetonitrile with 0.1% (v/v) formic acid were purchased from Scharlau. Ultrapure water (18.2 MΩ·cm resistivity at 25, < 10 ppb total organic carbon, passed through a 0.22–μm polyvinylidene difluoride filter) was generated from a Milli-Q Integral 5 water purification system.

#### Plant Cultivation and Harvesting

The sambong planting materials including the seedlings, garden soil, and pots were all obtained from Los Baños, Laguna, Philippines. All plants for the treatment experiment were obtained using the cutting method. After a rooting period of 50–60 d, healthy plants were transferred to 2-L pots containing garden soil. These plants were kept in the greenhouse for another 15–20 d to adapt and acclimatize. After this period, the plants were divided into 10 separate pots according to their respective treatments. The environmental and post-harvest processing parameters were randomized across the plant samples via a Plackett-Burman design (see Supplementary Table 1). Pots were placed either under sunlight or under a high-density polyethylene woven shade net (55–60% sun-shading). Pots were watered daily to maintain their respective soil moisture content, as indicated in Supplementary Table 1.

#### Sample Preparation and Liquid Chromatography

During harvest, the collected sambong leaves were washed with water, dried in a convection oven at 70°C for 5 h, ground, and stored at −20°C before use. Samples were extracted with either methanol (E1), ethyl acetate (E2), or n-hexane (E3). Each sample was prepared in six replicates. Extracts were filtered and passed through 0.2–μm polytetrafluoroethylene filters prior to LC analysis.

Ultra-high-performance liquid chromatography (UPLC) (Want et al., 2010) was performed using a Waters ACQUITY I-Class UPLC with ACQUITY photodiode array (PDA) eλ Detector. A reverse-phase Waters ACQUITY HSS C18 column (2.1-mm internal diameter ×100-mm length; 1.8–μm particle size) was used and maintained at 30°C. The mobile phases consisted of 0.1% formic acid in ultrapure water (A) and 0.1% formic acid in acetonitrile (B). A gradient elution was performed at a flow rate of 0.4 mL/min with an injection volume of 2 μL. The gradient was as follows: 20% B (0–3 min), 20–50% B (3–20 min), 50–100% B (20–22 min), 100–20% B (22–23 min), and 20% B (23–25 min). A PDA detector was used to scan the UV absorbance in the wavelength range of 200–700 nm and at a single wavelength channel of 285 nm. UV absorbances were acquired for only up to 20 min during the UPLC run time. A 40–μg/mL solution of naringenin was used as an external standard for relative quantification. All LC-UV data were acquired using MassLynx (Waters Corporation, Milford, USA).

### Pre-processing of the Input Data Sets

Although the REMS was programmed to collect data approximately every 2 ms, this automated data collection may be compromised due to power interruptions, as well as other logistical and hardware concerns. We applied a stochastic regression imputation (Wang and Oates, 2015) using a stochastic fitting function to fill in the missing values in any of the environmental data sets due to these logistical issues (see Supplementary Figure 3). For comparison, we used both the non-imputed and imputed input data sets for training the CNN model. Non-imputed input data are sensor data that contain missing values or NaNs due to interruptions in data collection. Imputed input data are those with missing values that have been replaced or imputed with stochastic variables.

Moreover, the environmental time-series data *X* = {*x*_1_, *x*_2_, …, *x*_*N*_} collected from the REMS must be normalized because it does not possess the same range as the output values. To achieve this, we applied technical indicators used in financial stock market chart analysis (Dash and Dash, 2016) such as William's R and stochastic oscillators to transform the range [0, 1] while preserving any seasonality trends and auto-regressive features in the time-series data. This normalization procedure resampled our initial observation *X*(*t*) in a uniform a set of $\tilde{X}\left(t\right)=\left\{\tilde{x_{1}},\;\tilde{x_{2}},\;\ldots ,\;\tilde{x_{N}}\right\},$ where each $\tilde{x_{i}}$ represents data collected every minute ranging between 0 and 1.

Finally, a tempo-spatial transformation (Wang and Oates, 2015; Fawaz et al., 2019) known as Gramian Angular Summation (Difference) Fields (GASF and GADF) was used to convert the resulting normalized time-series data to a 128 × 128 image (see Supplementary Figure 4). Upon resampling $\tilde{X}\left(t\right)$ to a 128-vector, each pixel in the resulting image therefore contains about 6 h of environmental data. As a result, *k* has an upper-bound value of $k_{\max}\;=\text{ ceil}\left(\frac{n}{128}\right)$ that can be used for data augmentation. We considered multiple combinations of *k* ∈ {5, 20, 30, 60, 720, 1440, 4320, 7200} min and *d* ∈ {720, 1440, 4320} min for these equations to increase the number of pairwise training data for the neural network by about 12-fold (see Supplementary Methods).

### Pre-processing of the Output Data Sets

The typical outputs for CNNs are in the range of [0, 1]. However, raw chromatographic data sets, specifically LC-UV data, have absorbance units that are above the order of 10^5^. Therefore, a pre-processing procedure must be conducted before training the CNN model. For instance, a min-max normalization could be applied to these output data sets to achieve the desired range. However, chromatograms are not free from noises and disturbances from the environment. Baseline drifts, for example, are caused by column or temperature changes during elution. As a result, min-max normalization could wrongly identify the minimum and maximum peak height of the signals with baseline drifts. Furthermore, the peak heights in the original chromatogram will not be preserved because the CNN model will only predict values in the range of [0, 1]. Peak heights are very important features of a chromatogram because they relate to the relative concentrations of different metabolites in the extract.

In this work, we first corrected the baseline drifts by using the BEADS algorithm (Ning et al., 2014). We then transformed the raw data *A*(*t*) to its relative concentration profile *C*(*t*) (see Supplementary Figure 5). The relative concentration of each metabolite was calculated as mg naringenin equivalents per 100 mg of dried leaves (mg/100 mg or %). The normalization of *C*(*t*) is derived from its area under the curve, which is basically the concentration of all of the detected metabolites in the extracts. The resulting normalized relative % concentrations *C*(*t*) will already be in the desired range of [0,1], but they are typically on the order of 10^−4^ and 10^−3^. This order of *C*(*t*) may lead to vanishing gradients and slow convergence during the training of the neural network. To mitigate these problems, we scaled up *C*(*t*) to the order of 10^−1^ by taking the log normalized % relative concentration y^*^(*t*). Because this log transformation has a unique inverse, the % concentration *C*(*t*) can be easily obtained from the predicted log concentration profile y^*^(*t*) of the model from any given sample.

### Input Data Types and the CNN Model

In CNNs, the input data can be generalized to a spatial data set or an image of the form *W* × *H* × *D*, where *W*, *H*, and *D* refer to the width, height, and depth of the input. In this work, we formed three types of input training data with varying depths: (1) non-imputed 1d data, (2) imputed 1d data, and (3) imputed 5d data (see Supplementary Figure 6).

Because we have a total of five environmental parameters to correlate per one output chromatogram of a sample extract, we horizontally concatenated each 128 × 128 image of the parameter to form input data with a depth of *D* = 1 (1d), or of the size 640 × 128 × 1. To compare the model performances achieved using different input data structures, we also stacked the five 128 × 128 images to form *D* = 5 (5d) input data with dimensions of 128 × 128 × 5. These three types of input data were trained separately using the same CNN model (see Supplementary Figure 7). The CNN is composed of four convolution layers for extracting pertinent features from the input images, as well as two fully-connected layers for correlating these features to the log relative % concentration profile of the samples. A total of 6,048 pairwise input-output data were obtained after performing data augmentation on the input data set. A model was trained using 85% of the pairwise data set (randomly selected) and evaluated using the remaining 15%. The metrics used for model evaluation were the cross-correlation, *R*^2^, and the Matthew's Correlation Coefficient (*MCC*) (Boughorbel et al., 2017). During training, we used the mean absolute error for the cost function and RMSProp for the optimization algorithm.

## Results

### Model Evaluation for the Different Input Data Types

**Figure 2:**
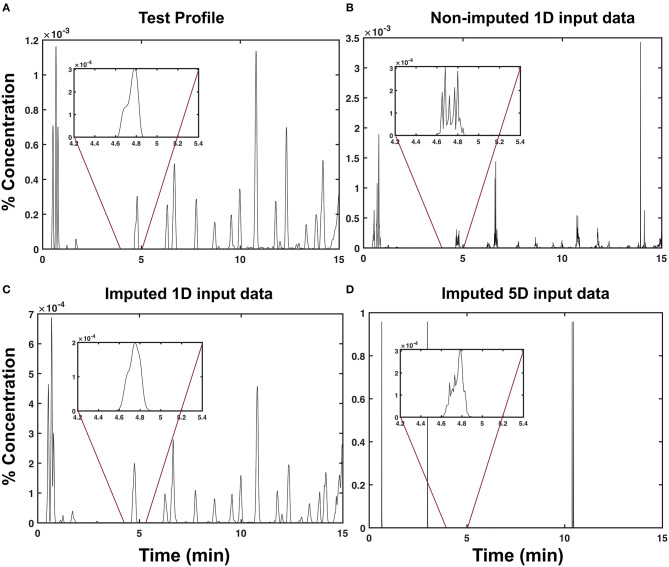
Predicted chromatograms of sambong leaf extracts. Sample chromatographic profiles (*C*(*t*) in % concentration) of a test extract obtained via **(A)** LC-UV chromatography, **(B)** model prediction using non-imputed 1d input data, **(C)** model prediction using imputed 1d input data, and **(D)** model prediction using imputed 5d input data.

An example of a predicted chromatogram produced using each input data type is shown in Figure 2. By inspection, Figure 2D is the least similar to the test chromatogram (Figure 2A) among the other input data types. Although it contains outliers beyond the 10^−3^ range of the test chromatogram (Figure 2A), it was still able to recover the peak located around *t* = 4.8 min, as shown in the inset plot. Among the three input data types, the imputed 1d input data type (Figure 2C) yielded the most visually similar profile, as shown in Figure 2A.

**Figure 3:**
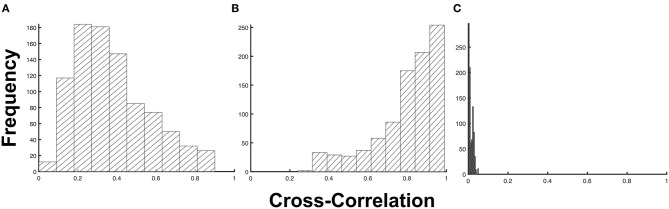
Frequency distribution of the cross-correlation metric. The cross-correlation distribution for **(A)** non-imputed 1d input data (*p* = 1.75 × 10^−4^), **(B)** imputed 1d input data (*p* = 1.09 × 10^−15^), and **(C)** imputed 5d input data (*p* = 0.89) at a 5% level of significance.

To generalize this observation, we measured the degree of similarity between the test and predicted profiles using a cross-correlation. As shown in Figure 3, the imputed 1d input data type obtained the highest average cross-correlation of μ_*xcorr*_ = 0.798 ± 0.163 (s.d). This indicates that the predictions from the model obtained using the imputed 1d input data have a high degree of similarity to the test samples. At the extreme end is the imputed 5d input data type, which demonstrated the lowest average cross-correlation of μ_*xcorr*_ = 0.013 ± 0.011 (s.d). This very low average cross-correlation can be attributed to the outliers observed in Figure 2D. If these points were to be filtered from the raw predictions of the model, the cross-correlation for the 5d input data will increase dramatically to μ_*xcorr*_ = 0.771 ± 0.170 (s.d).

**Figure 4:**
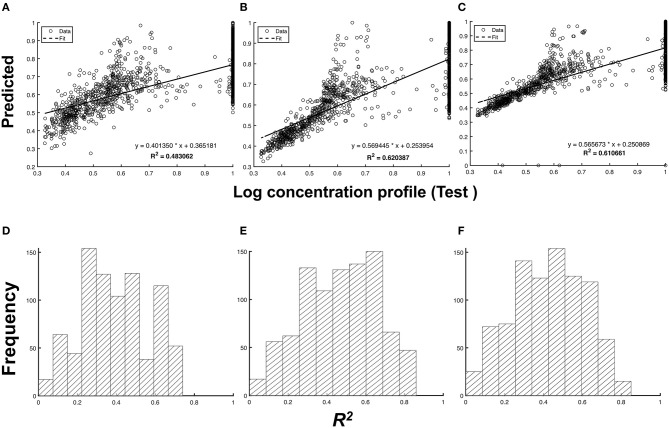
Comparison between the predicted and test chromatogram across different types of input data. Scatterplot of the log-transformed chromatogram, predicted vs. the test chromatogram: **(A)** non-imputed 1d input data, **(B)** imputed 1d input data, and **(C)** imputed 5d input data. The frequency distribution of *R*^2^ for all test samples for **(D)** non-imputed 1d input data (*p* = 1.36 × 10^−4^), **(E)** imputed 1d input data (*p* = 2.50 × 10^−6^), and **(F)** imputed 5d input data (*p* = 1.89 × 10^−5^) at a 5% significance level.

In Figure 4, we quantified the accuracy of the predictions by measuring the coefficient of determination, or *R*^2^, between the test and predicted profiles. Unlike the cross-correlation that measures the overall similarity of two signals based on their phase difference, the *R*^2^-value measures the accuracy of the predicted y^*^(*t*). We observed in Figures 4C,F that the predictions from the imputed 5d input data type have a higher mean *R*^2^ [$\mu_{R^{2}}\;=\;0.426\pm 0.183$ (s.d)] despite having the lowest μ_*xcorr*_ compared to the non-imputed input data type [$\mu_{R^{2}}\;=\;0.394\pm 0.173$ (s.d)]. This can be attributed to the presence of outliers in the predicted chromatograms. These outliers skew the resulting regression model away from the non-outlier data points, thereby increasing *R*^2^.

Interestingly, the model with 5d input data performed poorly compared to the model that uses 1d input data, despite both containing the same amount of temporal information from the sensor data. This suggests that the predictive performance of a model does not only depend on data integrity, but also on the structure of the input layer. More complicated structures of the input layer require complex combinations of filters and weights of the CNN. In 5d input data sets, two additional 128 × 128 images were stacked in addition to the usual 3d inputs (representing the RGB channels in images) for the CNN. A different architecture might be required to attain an equal or greater performance than achieved by the 1d inputs. Nonetheless, it can be observed from Figures 4B,E that using the imputed 1d input data type for the given neural network yields the most accurate predictions among the three input data types [$\mu_{R^{2}}\;=\;0.470\pm 0.192$ (s.d)].

### Peak Evaluation in the Predicted Profiles

**Figure 5:**
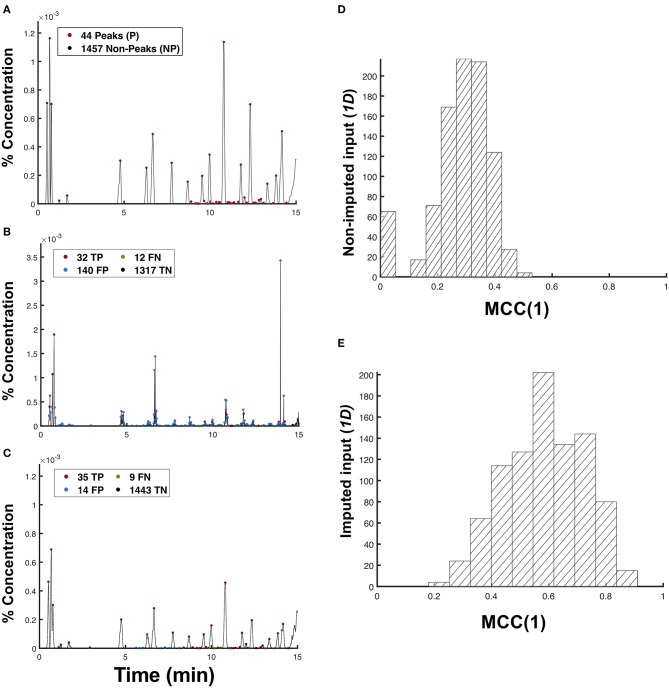
Matching of peaks between the predicted and test chromatogram. Peak classifications for *N* = 1, 501 points in the **(A)** test chromatogram, and the predictions from the **(B)** non-imputed and **(C)** imputed 1d input data types. The *MCC*(1) distribution for the **(D)** non-imputed input data (*p* = 4.50 × 10^−3^) and **(E)** imputed 1-channel input data type (*p* = 1.89 × 10^−5^) at a 5% significance.

The most important feature in a chromatogram is its peaks. A peak represents a metabolite, and the area under its curve is related to the concentration of that metabolite in the sample. To assess the performance of the CNN in terms of peak reconstruction and classification, we matched the peaks identified in the test and predicted chromatograms as shown in Figure 5. We only considered the predictions resulting from the 1d input data types because they both demonstrated a higher degree of similarity with the test chromatograms compared to that obtained using the 5d input data.

In matching the predicted peaks *p*′ with the test peaks ***P***, we first classify a predicted peak *p*′ as a true peak *tp* if it lies within a tolerance *nσ* of the test peak *p*. This peak tolerance also addresses the peak shifts that may have occurred during the chromatography procedure, thereby making this classification of predicted peaks robust to such disturbances. Mathematically, the set of true peaks *tp* can be expressed as:

(1) $$\begin{array}{l} tp=\{p^{\prime }\in P^{\prime }|\;p-n\sigma \;\leq p^{\prime }+n\sigma^{\prime }\leq p\\ +n\sigma ,\;\forall \;n\in Z,p\in P|\} \end{array}$$

where ±*nσ* is the peak tolerance and σ, σ′ are the standard deviations of the Gaussian curves approximated by the peaks *p* and *p*′, respectively. From this definition, we could also identify the false positive peaks *fp* as the set of predicted peaks *p*′ that do not correspond to any peaks in the test profiles (*fp* = {*p*′∈ ***P***′| *p*′+ *nσ*′ ∉ *tp*}). Conversely, false negatives are those non-peaks in the predicted profile that should have been classified as true peaks by the model (*fn* = {*np*′ ∈ ***NP***′ | *np*′ ∈ *tp*}), while true negatives are those non-peak points in both the predicted and test profiles (*tn* = {*np*′ ∈ ***NP***′ | *np*′ ∉ *tp*}). Using these definitions, we may evaluate the performance of the model in terms of the peak classification using Matthew's Correlation Coefficient (MCC) given by:

(2) $$\begin{array}{l} MCC\left(n\right)\\ =\frac{TP\times TN-FP\times FN}{\sqrt{\left(TP+FP\right)\times \left(TP+FN\right)\times \left(TP+FN\right)\times \left(TN+FP\right)}} \end{array}$$

where *TP*, *TN*, *FP*, and *FN* are the cardinality of the sets *tp, fp, tn*, and *fn*, respectively. An *MCC* of +1 implies a perfectly correct predictor; an *MCC* of 0 is as good as a random guess; and an *MCC* of −1 implies a perfectly wrong predictor. We used *MCC* to evaluate the performance of our model because the distribution of the peak types in a chromatogram is imbalanced.

In Figure 5, the model obtained using a non-imputed data input has a higher *TP* compared to the model obtained using an imputed data input. However, its *MCC*(1) = 0.3556 is significantly lower compared to the latter model with *MCC*(1) = 0.6736. This huge difference in *MCC*(1) is clearly a result of the presence of non-smooth peaks, as shown in Figure 2B. Because most peak detection algorithms function by using the first derivative test, those unwarranted sharp peaks in Figure 2B are classified as false positives peaks. The more false-positive or false-negative peaks that the model can classify, the lower its *MCC* value will be. This observation is evident in Figures 5D,E, wherein the non-imputed input data is shown to have obtained a significantly lower μ_MCC(1)_ = 0.283 ± 0.104 (s.d) compared to the imputed 1d input data type with μ_MCC(1)_ = 0.587 ± 0.138 (s.d).

As the peak classification hinges on the peak tolerance *n*σ, there exists a value *n* = n^*^ such that the increase of *MCC*(*n*) is no longer significant for *n* > n^*^. We considered the ratio *f*(*n*) as the basis of our optimization (Equation 3).

(3) $$\begin{array}{l} n^{*}=arg\;min\left\{f^{\prime }\left(n\right)|f\left(n\right)=\frac{\mu_{MCC}}{\sigma_{MCC}}\right\} \end{array}$$

**Figure 6:**
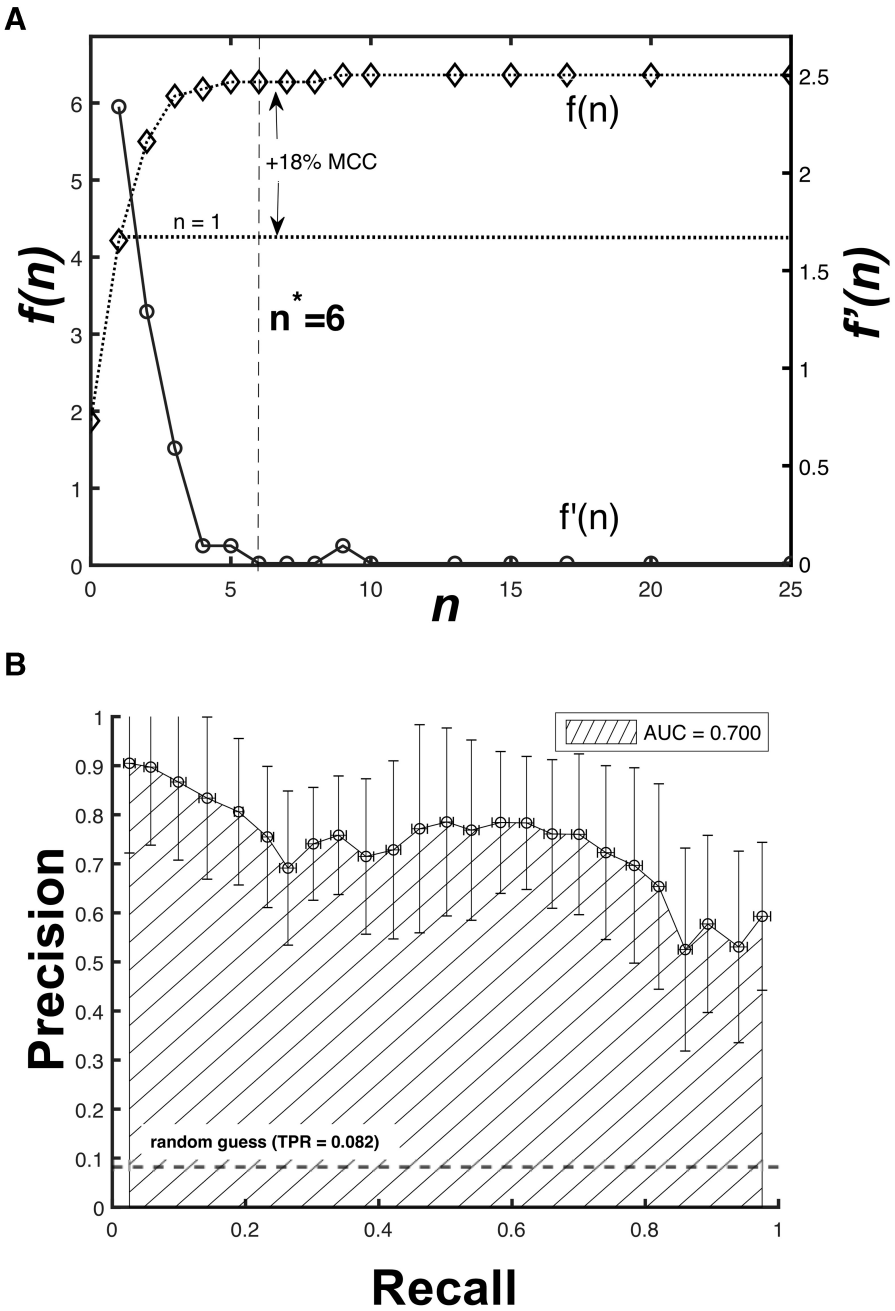
Optimization results for the peak tolerance and peak matching accuracy. **(A)** The rate of change *f*′(*n*) is monotonically decreasing for 1 ≤ *n* ≤ 6, which suggests an optimum value n^*^ = 6 resulting to an 18% improvement in *MCC*. **(B)** The Precision-Recall *AUC* curve for *MCC*(6) shows 70% accuracy of the CNN model.

The solution for Equation 3 is shown in Figure 6A. Although *f*(*n*) continues to increase for larger *n* values, the rate of change *f*′(*n*) is monotonically decreasing for 1 ≤ *n* ≤ 6, with *f*′(6) ≈ 10^−3^. This means that increasing *n* further corresponds to a diminishing gain in the peak classification accuracy. At the optimum value of n^*^ = 6, we obtained μ_*MCC*(6)_ = 0.691 ± 0.110 (s.d), which is a significant 18% improvement (*p* < 0.001, one-tailed *t*-test) from the previous mean we considered where μ_*MCC*(1)_ = 0.587 ± 0.138 (s.d).

Furthermore, we also cross-validated the model (60-fold) using different partitions of the imputed 1d input data set to obtain its overall performance. Figure 6B shows that the area under the curve (*AUC*) of the precision-recall plot for *MCC*(6) is equal to *AUC* = 0.70. A perfect classifier has an *AUC* = 1, suggesting that the CNN model is a sufficient predictor despite having only been trained with sensor data covering a month-long period. Increasing the amount of time over which a data set is available will further improve the predictive performance of the trained model.

### Model Performance for Different Solvents

**Figure 7:**
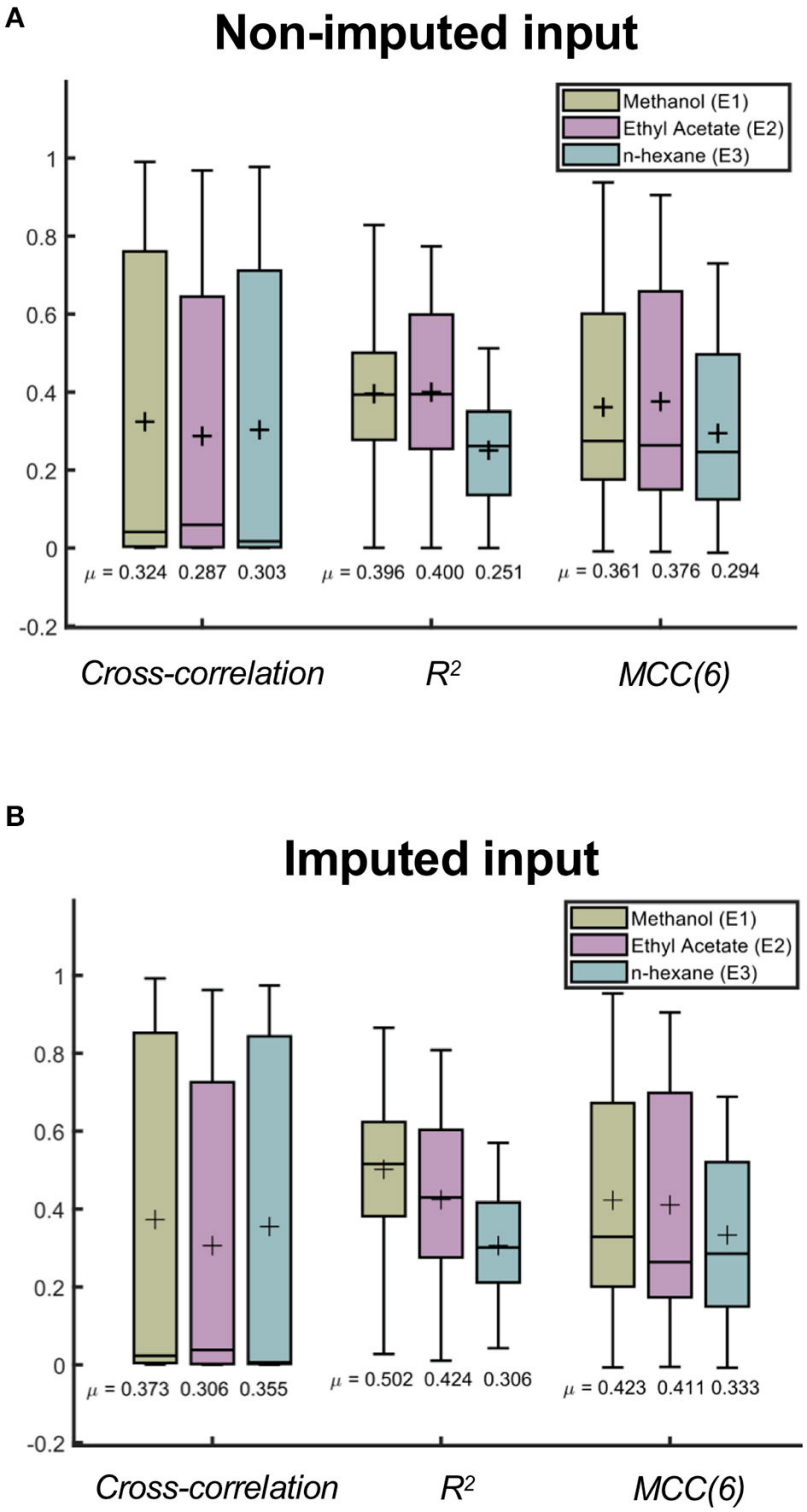
Comparing the model performance across three solvent systems used during extraction. Randomized cross-validation results (60 folds) of the CNN model for different solvent systems in **(A)** non-imputed (*E*1 = 24, 996 samples, *E*2 = 27, 147 samples, and *E*3 = 6, 877 samples) and **(B)** imputed (*E*1 = 19, 218 samples, *E*2 = 21, 060 samples, and *E*3 = 5, 122 samples) 1d data sets. The cross mark represents the average metric score of each distribution.

The appearance of the chromatogram is dependent on the solvent that is used to perform the extraction. In this work, the solvent system spans the extremes of dielectric constants, which could likely indicate that the chromatograms would represent subsets of very different extracted compounds. To determine if the model would perform better using a particular solvent system, we also assessed the model performance in relation to the type of solvent used in each sample. Figure 7 summarizes the differences among the three solvents after cross-validating the model using the non-imputed and imputed 1d data sets (k-folds = 60 folds). Consistently, the results of the cross-validation showed a better performance for the model that uses imputed 1d data (see Supplementary Table 2).

In Figure 7, we observe differences in the average metric scores among the three solvents. This suggests that the model has a preference toward a specific solvent system. In particular, methanol (E1) has been shown to have the highest average metric scores for the imputed input data. The difference observed between ethyl acetate and n-hexane using this input data is significant (*p* < 0.001, one-tailed *t*-test), which implies that this trained model will more accurately predict the chromatograms of extracts with methanol compared to those with ethyl acetate or n-hexane (see Supplementary Tables 3, 4). Considering the model obtained using non-imputed input data, the difference between the methanol and ethyl acetate solvents is also significant. However, the model's preference for the best solvent system is inconsistent as it fluctuates between these two solvents depending on the metric that is being considered (see Supplementary Tables 3, 5).

## Discussion

Typically, controllable laboratory variables, such as solvent systems and ratios, are studied and standardized when evaluating the phytochemistry and bioactivity of herbal extracts. However, the plants' phenotypic plasticity in response to stress and their environment can also add significant variability to the phytochemical make-up of raw herbal materials. This inherent variability in plant extracts caused by plant-environment interactions make the standardization of herbal formulations, and other plant therapeutics challenging. Here, we have demonstrated the feasibility of tracking the changes in the phytochemical profile of plant extracts based on real-time measurements of a few environment and extract-preparation variables. As a result, we predicted the chromatograms of the *Blumea balsamifera* leaf extracts using an imputation-augmented convolutional neural network (CNN) that uses the image-transformed temporal measurements of the variables.

The methods that we have established in this work involve many data pre-processing steps that are inspired from multiple scientific disciplines. To pre-process the input data, the following steps were involved: (1) stochastic imputation for the missing sensor values usually applied in statistics involving real world data; (2) tempo-spatial transformation of the time series using GASFs and GADFs that are conceptually equivalent to the Gramian matrix in linear algebra; and (3) data augmentation using technical indicators that are commonly applied in stock chart analysis. The amalgamation of these seemingly unrelated techniques is what allowed us to normalize and use time-series data as an input for the CNN model that conventionally utilizes only spatial data sets. Moreover, our results also showed the importance of these pre-processing procedures, particularly imputing missing sensor data to improve the accuracy of the neural network model. Overall, deep learning strategies such as CNNs depend not only on the amount, but also on the quality of information that can be extracted from the data sets.

Furthermore, our methods can also address the baseline and peak shifts that commonly appear in chromatograms due to column or temperature changes during elution. Corrections in the baseline shifts are learned by the model as the training data sets, in particular the output chromatograms, undergo pre-processing using the BEADS algorithm. The peak shifts, conversely, can be resolved by optimizing the peak tolerance for each predicted peak in the profile. Aside from the physical disturbances that occur during elution, chromatograms are also affected by the choice of solvent used during extraction. From our results, we showed that there is a significant difference in the accuracy of the predictions obtained using different solvent types that span the extremes of dielectric constants. More specifically, we found that the trained model could more accurately predict the chromatogram of extracts when methanol was used. We found that methanol has the highest dielectric constant of 33 at 20 compared to ethyl acetate (6.08) and n-hexane (1.89). Although the scope of this work focuses primarily on environmental forcing as it effects phytochemical synthesis, we have also demonstrated the extent of this method in providing insights about the effect of solvent types on the predicted phytochemical profile of the extracts.

This novel approach for predictive chromatography highly depends on the volume and veracity of the training data sets, which include both the environmental and the laboratory parameters. If trained with a sufficient amount of data, this method could provide an alternative high-throughput chromatography procedure for the identification and relative quantification of bioactive compounds for plant therapeutics. Unlike other methods used for the phytochemical profiling of plant extracts, the trained CNN model would only rely on the time-varying environmental data obtained for an area of vegetation. Without requiring any tedious laboratory procedures, this method would be able to accurately and rapidly predict the phytochemical profile of a particular plant extract using only the data collected by the REMS. In future studies, the author would recommend the use of a more comprehensive and robust environmental monitoring system for collecting data over longer cultivation periods to observe the effects of year-long seasonal patterns on the phytochemistry of sambong leaves.

Although the proposed technique used LC-UV chromatograms, it may also work with chromatograms generated by other spectral approaches, such as LC-MS. Furthermore, while we applied this method toward extracts taken from the leaf of a *Blumea balsamifera*, it is also possible to use the same framework for other plant species and for plant extracts from various parts of the plant, such as the root, seed, or fruit. For example, the same set of environmental time-series data could predict the chromatograms of extracts obtained from different plants or plant parts exposed to the same conditions, such as those present in a greenhouse.

Therefore, the proposed method may also function as an encoder of environmental forcing on phytochemistry across multiple herbal species. The encoder could be useful for controlling the growth and extract preparation conditions to intensify the expression of specific bioactive compounds, or the combinations thereof, in the extracts. The method can also detect the impact of climate change in the form of significant structural modifications to the phytochemical compositions of plant species over extended periods of time.

Moreover, the proposed method may also be used to discover previously unknown metabolites that contribute to the observed therapeutic effects of the herbal extracts. This accurate and high-throughput alternative to the tedious laboratory and chromatographic procedures could permit the fast screening of putative bioactive compounds across multiple herbal species. Therefore, the synthesis of both herbal formulations or single-molecule medicines could become much faster.

Another practical application of the proposed method is quality-assurance verification at the phytochemical level for plant-derived produce from farms. An automated system for identifying which environmental factors exert a significant impact on plant phytochemistry could provide valuable insights for optimizing produce characteristics. For example, farmers could use such insights to enhance their current farming practices to increase production and improve quality control of their products. Although in this study we saw the limitations of our method in terms of continuous power supply and robust sensing instrumentation, we believe that the fast pace technology advancement would address these limitations and enhance the practicability of our method, especially in the actual farm setting.

Lastly, this framework that may be used to attribute the environment's influence on a plant's ability to synthesize compounds will be useful in the analytical chemistry of natural products in the future. It provides a direct and scalable means to encode complex environmental influences on the chemical synthesis processes within a plant. This framework is direct because it considers the impact of a combination of multiple environmental factors simultaneously without referencing any particular molecular theory of forcing. It is scalable because the method could assimilate additional environmental factors to obtain more accurate and precise predictions.

## Data Availability Statement

The original contributions presented in the study are included in the article/Supplementary Material, further inquiries can be directed to the corresponding author/s.

## Author Contributions

DJ conceptualized and designed the study. JB performed the experiments and simulations. Both authors analyzed and interpreted the data and wrote the manuscript.

## Conflict of Interest

The authors declare that the research was conducted in the absence of any commercial or financial relationships that could be construed as a potential conflict of interest.

## Publisher's Note

All claims expressed in this article are solely those of the authors and do not necessarily represent those of their affiliated organizations, or those of the publisher, the editors and the reviewers. Any product that may be evaluated in this article, or claim that may be made by its manufacturer, is not guaranteed or endorsed by the publisher.
