# Supplementary material for: Predictive Chromatography of Leaf Extracts Through Encoded Environmental Forcing on Phytochemical Synthesis
Source: Front Plant Sci. 2021 Aug 25;12:613507. doi: 10.3389/fpls.2021.613507 (PMC8424046; doi:10.3389/fpls.2021.613507)
Supplement: Supplementary file 1 [file Image_1.pdf]

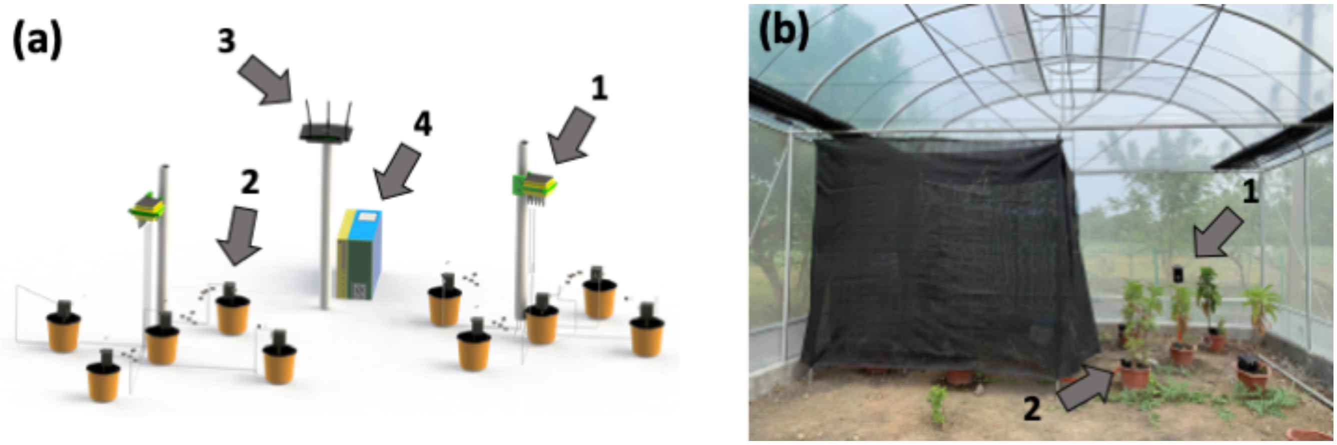

Supplementary Figure 1. The remote environmental monitoring system (REMS) in Los Baños, Laguna. The **(a)** schematic diagram, and the **(b)** actual setup of the REMS**.** This is the actual prototype of the REMS we built in the laboratory using microcontrollers and off-the-shelf sensors. Inset arrows: (1) the instrument for collecting global environmental parameters e.g. ambient light, temperature and humidity, (2) the instrument for collecting local environmental parameters i.e. soil moisture and soil pH, (3) router and (4) server for data collection. A high-density polyethylene woven shade net (55-60% sun shading) was used to cover the five pots. The rest of the pots was left untreated with the woven shade and thus, received natural lighting from the sun inside the greenhouse.
