# Supplementary material for: Predictive Chromatography of Leaf Extracts Through Encoded Environmental Forcing on Phytochemical Synthesis
Source: Front Plant Sci. 2021 Aug 25;12:613507. doi: 10.3389/fpls.2021.613507 (PMC8424046; doi:10.3389/fpls.2021.613507)
Supplement: Supplementary file 2 [file Image_2.pdf]

frontiers

log_date	Moisture	Light	Humidity	Temperature	pH
01/07/2019 13:40		1235.75	99.90000153	32.09999847	
01/07/2019 13:40					7.362309933
01/07/2019 13:40	86.6359024				
01/07/2019 13:40		1226.119995	99.90000153		
01/07/2019 13:40				32.09999847	7.306650162
01/07/2019 13:40	86.6359024				
01/07/2019 13:40		1228.72998	99.90000153		
01/07/2019 13:40				32.09999847	7.250989914
01/07/2019 13:40	86.6359024	1228.72998			
01/07/2019 13:40			99.90000153	32.09999847	
01/07/2019 13:40					7.195330143
01/07/2019 13:40	86.6359024	1228.72998	99.90000153		
01/07/2019 13:40				32.09999847	7.334479809
01/07/2019 13:40	90.35980225	1232.810059			
01/07/2019 13:40			99.90000153	32.09999847	7.362309933
01/07/2019 13:40	86.6359024				
01/07/2019 13:40		1238.359985	99.90000153		
01/07/2019 13:40				32.09999847	7.306650162
01/07/2019 13:40	86.6359024				
01/07/2019 13:40		1238.359985	99.90000153		
01/07/2019 13:40				32.09999847	
01/07/2019 13:40					7.362309933
01/07/2019 13:40	84.77400208				
01/07/2019 13:40		1247.98999			
01/07/2019 13:40			99.90000153	32.09999847	
01/07/2019 13:40					7.167510033
01/07/2019 13:40	84.77400208	1261.699951			
01/07/2019 13:40			99.90000153	32.09999847	
01/07/2019 13:40					7.306650162
01/07/2019 13:40	79.1882019	1269.859985			
01/07/2019 13:40			99.90000153		
01/07/2019 13:40				32.09999847	
01/07/2019 13:40					7.334479809

Supplementary Figure 2. A sample snippet of the environmental data from REMS. Each column represents an environmental variable collected by a sensor every two milliseconds. The data collection by different sensors is independent from one another, and thus the presence of blank spaces across the comma-separated values (CSV) file. The REMS collected these environmental data over one-month study period (May-June,2019).
