# Supplementary material for: Predictive Chromatography of Leaf Extracts Through Encoded Environmental Forcing on Phytochemical Synthesis
Source: Front Plant Sci. 2021 Aug 25;12:613507. doi: 10.3389/fpls.2021.613507 (PMC8424046; doi:10.3389/fpls.2021.613507)
Supplement: Supplementary file 3 [file Image_3.pdf]

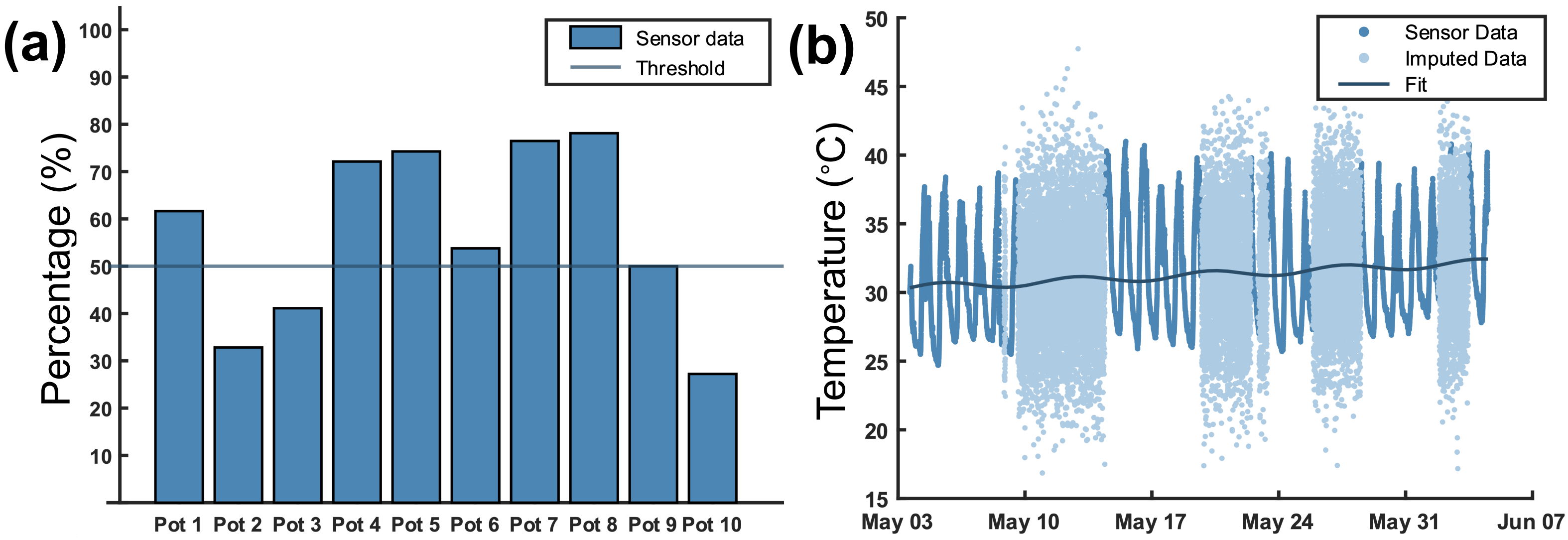

Supplementary Figure 3. Collection of sensor data by the REMS. **(a)** Six out of 10 pots contain sensor data that is above the 50% threshold. **(b)** A sample temperature data collected from Pot 1. We only used the data collected from the six pots (Pots 1,4,5,6,7 and 8) that have more than 50% sensor data so that the effect of noisy data will be reduced during the training. The sample result of the stochastic imputation to the missing values in temperature data from Pot 1 is also shown. Despite having a loss of data from the REMS due to logistical concerns, we were still able to preserve the seasonality that we expect from the environmental parameters using stochastic imputation.
