# Supplementary material for: Predictive Chromatography of Leaf Extracts Through Encoded Environmental Forcing on Phytochemical Synthesis
Source: Front Plant Sci. 2021 Aug 25;12:613507. doi: 10.3389/fpls.2021.613507 (PMC8424046; doi:10.3389/fpls.2021.613507)
Supplement: Supplementary file 4 [file Image_4.pdf]

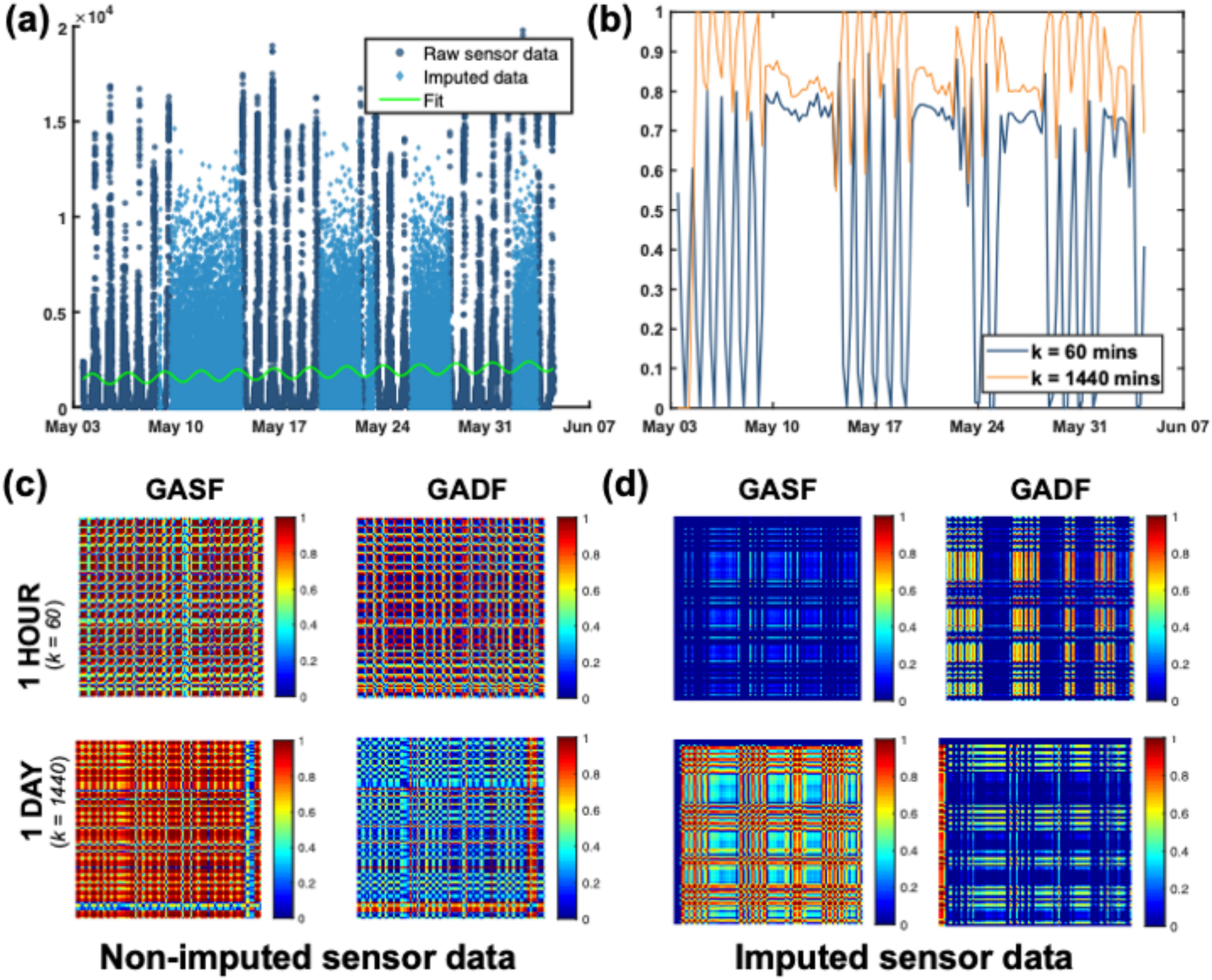

Supplementary Figure 4. The pre-processing of input data. This example shows the pre-processing procedure done to the ambient light data in Pot 1, which includes: **(a)** filling in missing raw sensor values using stochastic regression imputation, **(b)** normalizing time-series using technical indicators, **(c) - (d)** tempo-spatial encoding of normalized time-series data using Gramian Angular Summation/Difference Field (GASF/GADF). Using technical indicators such as William's R, we were able to preserve the trends in the normalized time-series dataset.
