# Supplementary material for: Predictive Chromatography of Leaf Extracts Through Encoded Environmental Forcing on Phytochemical Synthesis
Source: Front Plant Sci. 2021 Aug 25;12:613507. doi: 10.3389/fpls.2021.613507 (PMC8424046; doi:10.3389/fpls.2021.613507)
Supplement: Supplementary file 5 [file Image_5.pdf]

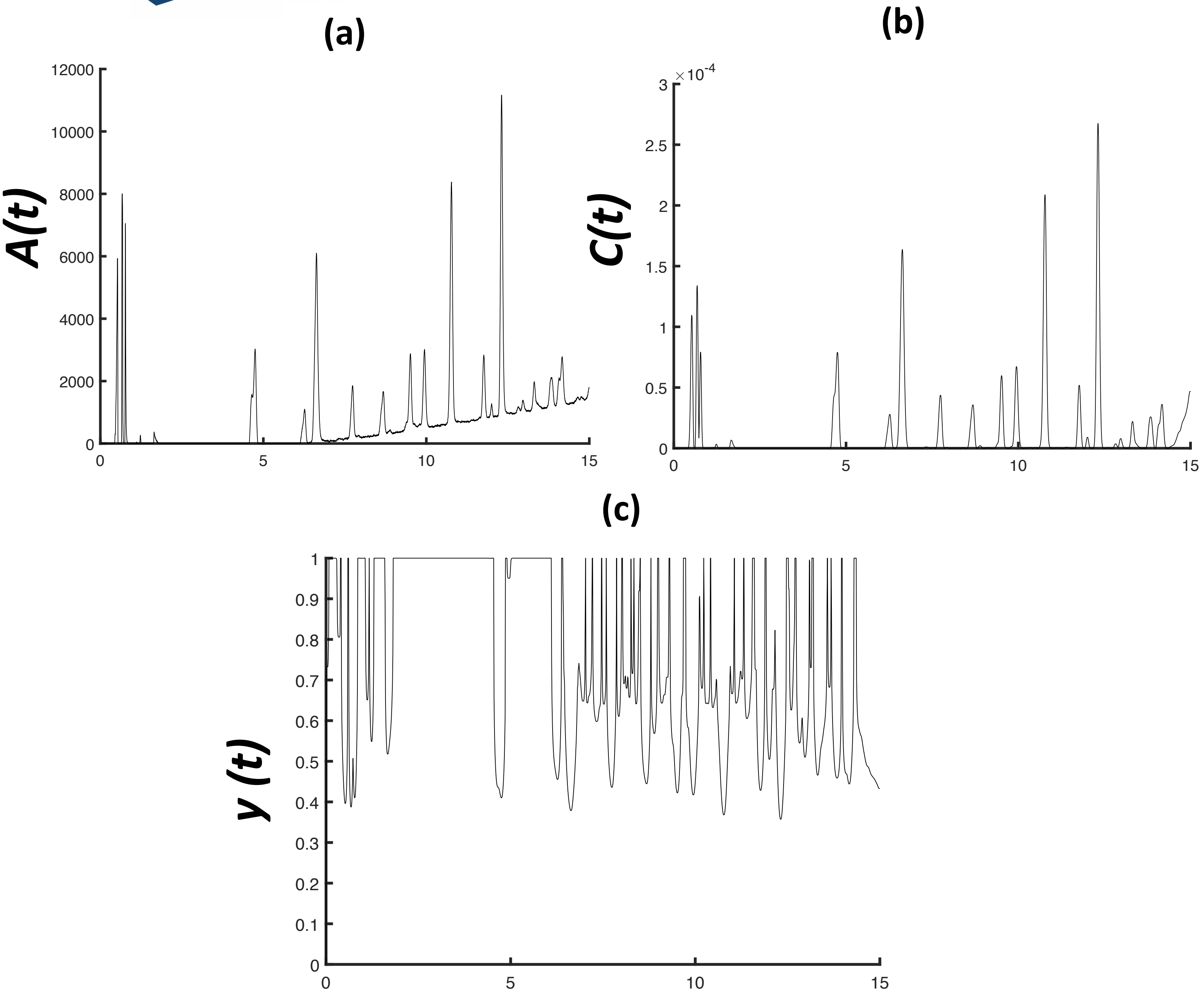

Supplementary Figure 5. The pre-processing of output data. The process of transforming **(a)** the raw chromatogram $\boldsymbol{A(t)}$ obtained from the LC-UV spectroscopy to its **(b)** concentration profile $\boldsymbol{C(t)}$, which was further normalized in **(c)** log concentration units for the training of the neural network. Initially, the raw signals which contain unwarranted baseline drifts are corrected using the BEADS algorithm. Although $\boldsymbol{C(t)}$ is already within the desired range of [0,1], this relative % concentration is typically between the order of $\mathbf{10^{-4}}$ and $\mathbf{10^{-3}}$ which may lead to vanishing gradients during the training of the neural network. A logarithmic transformation was applied to $\boldsymbol{C(t)}$ to scale up the order to $\mathbf{10^{-1}}$, which is a favourable output for the neural network. Since each transformed chromatogram has a total retention time of 15 minutes, every node in the output layer of the network is equivalent to $\boldsymbol{\Delta t = 0.01\ min}$ (total output nodes = 1501).
