# Supplementary material for: Predictive Chromatography of Leaf Extracts Through Encoded Environmental Forcing on Phytochemical Synthesis
Source: Front Plant Sci. 2021 Aug 25;12:613507. doi: 10.3389/fpls.2021.613507 (PMC8424046; doi:10.3389/fpls.2021.613507)
Supplement: Supplementary file 6 [file Image_6.pdf]

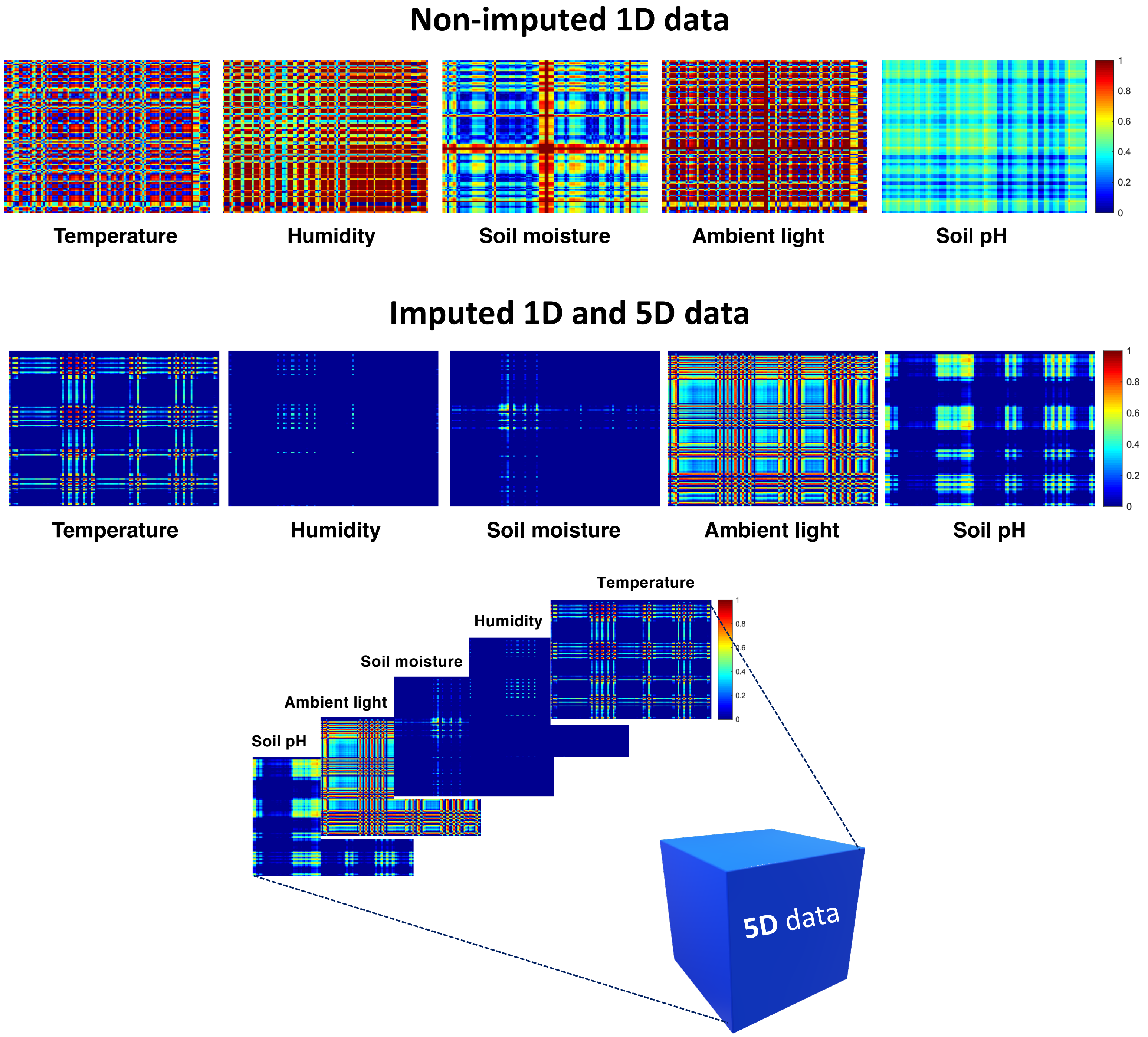

Supplementary Figure 6. Sample of a training image for different input data types. The input layer of the CNN model accepts a variable input size of 128x128xD, where D may be equal to 1 or 5. For 1D input type, the images (128x128 pixels) of the five environmental parameters are concatenated horizontally while for 5D, the images are concatenated vertically. The order of concatenations as shown above, is preserved all throughout the training and evaluation of the CNN model.
