# Supplementary material for: Predictive Chromatography of Leaf Extracts Through Encoded Environmental Forcing on Phytochemical Synthesis
Source: Front Plant Sci. 2021 Aug 25;12:613507. doi: 10.3389/fpls.2021.613507 (PMC8424046; doi:10.3389/fpls.2021.613507)
Supplement: Supplementary file 7 [file Image_7.pdf]

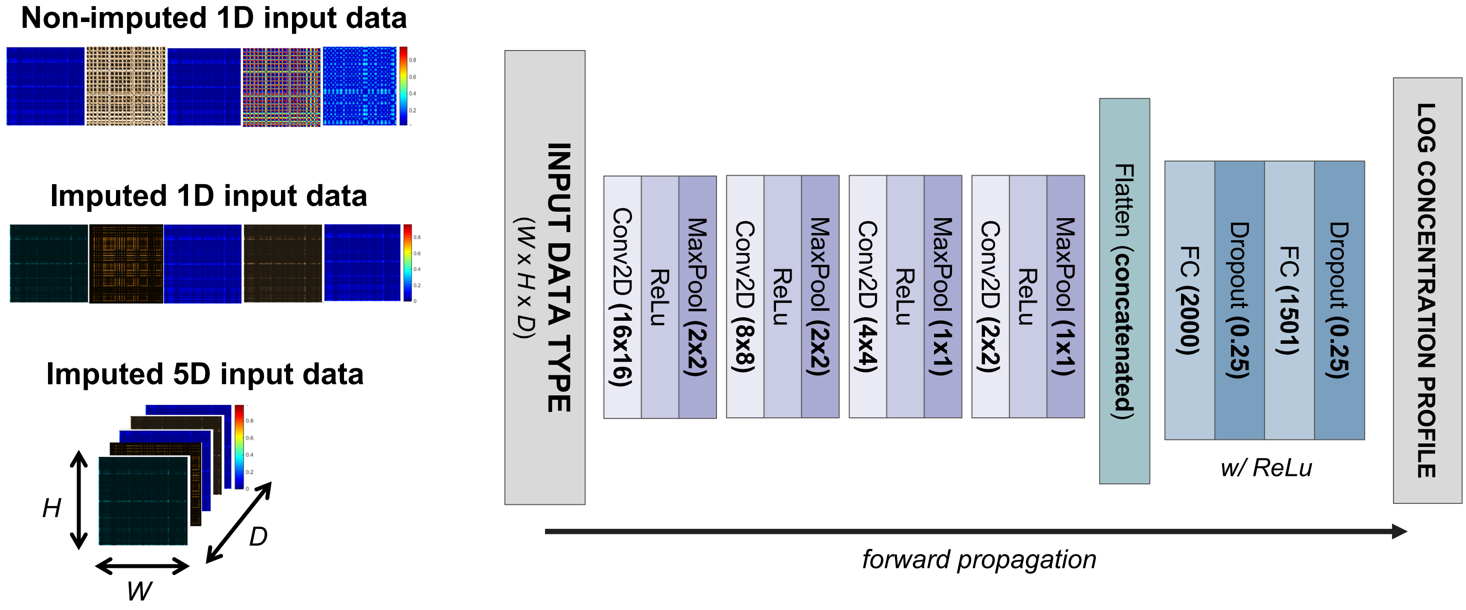

Supplementary Figure 7. The CNN model and different input data types. The CNN models for different input data types share the same architecture except for the input layer. For 1D input data, the input layer comprises of 640x128x1 input nodes, while there are 128x128x5 input nodes for the imputed 5D input data. In the output layer, a softmax function was used. Furthermore, all the models have a total of 1501 nodes in their output layer, where each node is equivalent to $\boldsymbol{\Delta t} = \mathbf{0.01}\ \boldsymbol{min}$ of the chromatogram. A total of 6048 pairwise input-output data was obtained after performing data augmentation in the input dataset. During training, we used the mean absolute error for the cost function, and the RMSProp for the optimization algorithm. The choice of CNN for this study was inspired by its recent successes in the field of computer vision. This also allowed us to study and explore the architectures from the existing models that may be useful in solving our problem.
