# Supplementary material for: Predictive Chromatography of Leaf Extracts Through Encoded Environmental Forcing on Phytochemical Synthesis
Source: Front Plant Sci. 2021 Aug 25;12:613507. doi: 10.3389/fpls.2021.613507 (PMC8424046; doi:10.3389/fpls.2021.613507)
Supplement: Supplementary file 8 [file Table_1.pdf]

Supplementary Table 1. The Plackett-Burman design of experiment.

Sample No.	Pot No.	Light Intensity	Soil Moisture	Drying Temperature	Solvent Polarity	Solvent-Sample Ratio	No. of Replicates
1	1	Shaded	70% WC	70 °C	E2	50:1	6
2	2	Shaded	30% WC	70 °C	E1	50:1	6
3	3	Shaded	70% WC	70 °C	E2	40:1	6
4	4	Shaded	50% WC	70 °C	E1	50:1	6
5	5	Shaded	30% WC	70 °C	E1	40:1	6
6	6	Full	30% WC	70 °C	E2	40:1	6
7	6	Full	30% WC	70 °C	E3	30:1	6
8	7	Full	30% WC	70 °C	E2	50:1	6
9	8	Full	70% WC	70 °C	E1	40:1	6
10	9	Full	70% WC	70 °C	E3	30:1	6
11	9	Full	70% WC	70 °C	E1	30:1	6
12	9	Full	70% WC	70 °C	E2	30:1	6
13	10	Full	50% WC	70 °C	E3	50:1	6
14	10	Full	50% WC	70 °C	E1	30:1	6
15	10	Full	50% WC	70 °C	E2	30:1	6
