# Supplementary material for: Predictive Chromatography of Leaf Extracts Through Encoded Environmental Forcing on Phytochemical Synthesis
Source: Front Plant Sci. 2021 Aug 25;12:613507. doi: 10.3389/fpls.2021.613507 (PMC8424046; doi:10.3389/fpls.2021.613507)
Supplement: Supplementary file 9 [file Table_2.pdf]

Supplementary Table 2. Cross-validated results for different 1D input data types

1D Input data	Mean and standard deviation *(k-folds=60)*		
	Cross-correlation	R^2	MCC(6)
**Imputed**	$\mathbf{0.340 \pm 0.395}$	$\mathbf{0.444 \pm 0.187}$	$\mathbf{0.407 \pm 0.260}$
**Non-Imputed**	0.305 ± 0.345	0.381 ± 0.172	0.360 ± 0.252

Supplementary Table 3. Cross-validated results for different solvent system

1D input data	Mean and standard deviation *(k-folds=60)*								
	Cross-correlation			R^2			MCC(6)		
	E1	E2	E3	E1	E2	E3	E1	E2	E3
**Imputed**	$\mathbf{0.373 \pm 0.421}$	0.306 ± 0.361	0.355 ± 0.412	$\mathbf{0.502 \pm 0.175}$	0.424 ± 0.188	0.306 ± 0.124	$\mathbf{0.423 \pm 0.251}$	$0.411 \pm .277$	0.333 ± 0.195
**Non-imputed**	$\mathbf{0.324 \pm 0.376}$	0.287 ± 0.330	0.303 ± 0.367	$\mathbf{0.396 \pm 0.148}$	0.400 ± 0.189	0.251 ± 0.112	0.361 ± 0.237	$\mathbf{0.376 \pm 0.272}$	0.294 ± 0.203

Suppl. Table 4. Significance test for solvent system analysis with imputed 1D input data

	p-values (alpha = 5%)								
	Cross-correlation			R^2			MCC(6)		
	E1	E2	E3	E1	E2	E3	E1	E2	E3
**E1**	0	0.007	< 0.001	0	< 0.0001	< 0.0001	0	< 0.0001	< 0.0001
**E2**	0.007	0	< 0.0001	< 0.0001	0	< 0.0001	< 0.0001	0	< 0.0001
**E3**	< 0.001	< 0.0001	0	< 0.0001	< 0.0001	0	< 0.0001	< 0.0001	0

Suppl. Table 5. Significance for solvent system analysis with non-imputed 1D input data

	p-values (alpha = 5%)								
	Cross-correlation			R^2			MCC(6)		
	E1	E2	E3	E1	E2	E3	E1	E2	E3
**E1**	0	0.007	< 0.001	0	0.006	< 0.0001	0	< 0.0001	< 0.0001
**E2**	< 0.0001	0	0.0004	0.006	0	< 0.0001	< 0.0001	0	< 0.0001
**E3**	< 0.001	0.0004	0	< 0.0001	< 0.0001	0	< 0.0001	< 0.0001	0
