# Supplementary material for: Predictive Chromatography of Leaf Extracts Through Encoded Environmental Forcing on Phytochemical Synthesis
Source: Front Plant Sci. 2021 Aug 25;12:613507. doi: 10.3389/fpls.2021.613507 (PMC8424046; doi:10.3389/fpls.2021.613507)
Supplement: Supplementary file 10 [file Presentation_1.pdf]

1 Supplementary Methods

1.1 Normalization and imputation of input data

Although the REMS was programmed to collect every two milliseconds, this automated data collection may be compromised due to power interruptions, and other logistics and hardware concerns. As a result, the value per pixel in the image may contain NaN values. In order to ensure the integrity of our sensor data, we filled in those missing values using a stochastic fitting function of the form:

$$X'(t) = C_1 \sin C_2 t + C_3 t + C_4 + \chi(\mu, \sigma) \tag{1}$$

where χ is a Gaussian white noise with μ and σ equal to the respective mean and standard deviation of the residual $r = |X'(t) - X(t)|$. We used Levenberg–Marquardt algorithm (LMA) to solve for the parameters C_1, C_2, C_3 and C_4 in Equation (1).

The time-series environmental data $X = \{x_1, x_2, \ldots, x_N\}$ collected by the sensors have to be normalized first since they do not share the same range of output values. We applied technical indicators used in financial stock market chart analysis such as William's R and stochastic oscillators to transform their range [0,1] while preserving any seasonality trends and auto-regressive features in the time-series data. Since the sensors transmit data every second, we calculated the low, open, and closing sensor values every minute, as in the context of stock chart market on a trading period. This resampled our initial observation X to a uniform a set of $\tilde{X}(t) = \{\widetilde{x_1}, \widetilde{x_2}, \ldots, \widetilde{x_N}\}$, where each $\widetilde{x_i}$ represents minutely information from the sensors. From this uniform, temporal sequence of data, we can obtain the William's R using the following equation:

$$\boldsymbol{WR}^k\,(t) = \frac{\boldsymbol{HH_t}^k - \boldsymbol{C_t}^k}{\boldsymbol{HH_t}^k - \boldsymbol{LL_t}^k} \tag{2}$$

where $\boldsymbol{HH_t}^k, \boldsymbol{LL_t}^k$ and $\boldsymbol{C_t}^k$ are the highest high, lowest low and closing sensor values within the past k period. For $t < k$, we let the values remain as $\boldsymbol{WR}^1(t)$. Similarly, for stochastic oscillators, the corresponding transformations are given by Equations (3) and (4), which represent the slow and fast oscillators, respectively.

$$\boldsymbol{SSTO}^k(t) = \frac{\boldsymbol{C_t}^k - \boldsymbol{LL_t}^k}{\boldsymbol{HH_t}^k - \boldsymbol{LL_t}^k} \tag{3}$$

$$\boldsymbol{FSTO_k}^d(t) = \frac{1}{d}\sum_{n=0}^{d-1} \boldsymbol{SSTO}^k(t-n) \tag{4}$$

1.2 Tempo-spatial transformation of environmental datasets

After pre-processing the raw sensor data, each environmental time-series contains more than 43,200 datapoints. However, due to power failures and other logistical concerns, the time-series data collected by the REMS contain NaNs or missing values. We only used the data collected from the six pots (Pots 1,4,5,6,7 and 8) that have more than 50% sensor data so that the effect of noisy data will be reduced during the training (see Supplemental Fig. S3). By performing data

augmentation to the collected data, we obtained a total of 6048 pairwise input-output dataset for the neural network training and evaluation.

Supplemental Fig. S4 shows a sample result of the tempo-spatial transformation using GASF and GADF. Each pixel in this image contains about 6-hour worth of environmental data from the raw time-series data. If the missing values were not to be imputed, the temporal resolution per pixel of the images would not be consistent, and it could result to a pixel containing NaNs or zero values. The difference between non-imputed and imputed input data is also shown in Supplemental Fig S4. Unlike the image of non-imputed 1D data, the patterns in the imputed datasets are periodic and pronounced within definite intervals. This reinforces the encoding of the auto-regressive information from the environmental time-series data to the spatially organized input datasets of the CNNs.

1.3 Determination of relative concentration profile from chromatographic datasets

We used the Equation (5) to derive the normalized relative concentration profile $C(t)$ of each sample from its raw chromatographic data $A(t)$.

$$C(t) = \beta \times A(t) \tag{1}$$

The factor β for each sample is determined using the following equation:

$$\beta = \frac{1}{AUC} \times \frac{r_s}{C_s} \times \frac{V_i}{W_x} \times \mathrm{D} \times \mathrm{R} \times \mathrm{F} \tag{6}$$

where r_s is the average signal response of the external standard solution (naringenin), C_s is the actual concentration of the external standard solution (in µg/mL), W_x is the weight of the leaves samples used for the analysis (in g), V_i is the volume where the samples were soaked (in mL), D is the dilution factor of the sample solution, R is the relative response factor between the peak compound and naringenin which, in this case, is assumed to be equal to 1.0, F is the conversion factor to change units to mg/100 mg or %, equivalent to 0.0001, and AUC is the area under the curve of $A(t)$.

Equation (5) gives us the desired range of [0,1]. However, the normalized % relative concentration profile of a sample is usually in the order of 10^{-3} or 10^{-4}. During backpropagation, this could lead to vanishing gradients. As a result, the convergence of the training algorithm would be slower. Also, we observed poor predictive performance of the CNN model when using $C(t)$ alone. So to further scale up the order of $C(t)$, we applied a logarithmic transformation given by

$$y^*(t) = \frac{\log(C + \varepsilon)}{\log(\varepsilon)} \tag{7}$$

where $\varepsilon = 10^{-10}$ is the machine precision. The resulting values are in the range of [0,1] and order 10^{-1}. These pre-processed outputs $y^*(t)$ will be paired with the pre-processed inputs of the environmental datasets for the CNN training. During evaluation, the CNN model will predict $y^*(t)$ from the given input images. Since Equation (7) has a unique inverse, we could easily obtain the concentration profile $C(t)$ from the predicted $y^*(t)$ of the model from any given sample.

1.4 Computing environment for the CNN training and evaluation

Many deep learning frameworks are available in different computer languages nowadays. There are really no specific advantages over the others, but we were most familiar with Python, and its open-source neural-network library called Keras. In this work, we used Keras 2.2.4 with Tensorflow-GPU 1.14 as the backend, for training and evaluating the neural network.

Since deep learning methods take time in exploring the best parameters and hyperparameters for the model, we utilized our two available machines that are equipped with GPU. We performed the same computations for different input types in an Intel® Core™ i7-7700 CPU with NVIDIA GeForce RTX 2080 Ti, and an Intel® Xeon® Gold 6152 CPU with NVIDIA GRID P40. Although comparison in the computational speed was beyond the scope of this work, we noticed a faster runtime (2x) in the NVIDIA RTX 2080 Ti environment compared to that of NVIDIA GRID P40.

Our choice of parameters and hyperparameters is completely based on trial and error, as with any studies in the deep learning field. Aside from the reported parameters (e.g. layers and optimizers) in our CNN model, we also used a batch size of 64 and an initial learning rate of 1e-6.
